# PO2RDF: representation of real-world data for precision oncology using resource description framework

**DOI:** 10.1186/s12920-022-01314-9

**Published:** 2022-07-30

**Authors:** Yiqing Zhao, Anastasios Dimou, Feichen Shen, Nansu Zong, Jaime I. Davila, Hongfang Liu, Chen Wang

**Affiliations:** 1 Division of Digital Health Sciences, Mayo Clinic, Rochester, MN USA; 2 Division of Medical Oncology, Department of Oncology, Mayo Clinic, Rochester, MN USA; 3 Department of Quantitative Health Sciences, Mayo Clinic, Rochester, MN USA; 4 Department of Mathematics, Statistics and Computer Science, St. Olaf College, Northfield, MN USA

**Keywords:** Resource description framework, Precision oncology, Electronic health records, Real-world evidence

## Abstract

**Background:**

Next-generation sequencing provides comprehensive information about individuals’ genetic makeup and is commonplace in precision oncology practice. Due to the heterogeneity of individual patient’s disease conditions and treatment journeys, not all targeted therapies were initiated despite actionable mutations. To better understand and support the clinical decision-making process in precision oncology, there is a need to examine real-world associations between patients’ genetic information and treatment choices.

**Methods:**

To fill the gap of insufficient use of real-world data (RWD) in electronic health records (EHRs), we generated a single Resource Description Framework (RDF) resource, called PO2RDF (precision oncology to RDF), by integrating information regarding genes, variants, diseases, and drugs from genetic reports and EHRs.

**Results:**

There are a total 2,309,014 triples contained in the PO2RDF. Among them, 32,815 triples are related to Gene, 34,695 triples are related to Variant, 8,787 triples are related to Disease, 26,154 triples are related to Drug. We performed two use case analyses to demonstrate the usability of the PO2RDF: (1) we examined real-world associations between EGFR mutations and targeted therapies to confirm existing knowledge and detect off-label use. (2) We examined differences in prognosis for lung cancer patients with/without TP53 mutations.

**Conclusions:**

In conclusion, our work proposed to use RDF to organize and distribute clinical RWD that is otherwise inaccessible externally. Our work serves as a pilot study that will lead to new clinical applications and could ultimately stimulate progress in the field of precision oncology.

## Background

Advancement in next-generation sequencing technologies and lowered testing costs have contributed to a much wider embracement of Precision Oncology [1] in oncology clinical practice. The potential of Precision Oncology is to enable oncologist practitioners to make better clinical decisions by incorporating individual cancer patients’ genomic information and clinical characteristics. The anticipation of Precision Oncology is to improve the selection of targeted therapies, avoid side effects from ineffective or toxic therapies, and therefore reduce healthcare costs while improving patient outcomes [2–5].

With increasing needs for Precision Oncology knowledge and evidence, specialized knowledgebases such as OncoKB [6], CIViC [7] and other more general pharmacogenomics or Precision Medicine knowledgebases include PharmGKB [8] and ClinVar [9] were established to curate comprehensive scientific evidence on genes, mutations, drugs, their combined effects on diseases or phenotypes. OncoKB annotates the oncogenic effects and clinical significance of somatic variants [6]. To date, it has curated 5293 unique mutations in 628 cancer-associated genes and 54 tumor types with 92 associated treatment options. Levels of evidence were evaluated based on evidence sources that ranged from US Food and Drug Administration (FDA) labeling, National Comprehensive Cancer Network guidelines, disease-focused expert group recommendations, and scientific literature [6]. OncoKB provides 300 mutation-treatment associations that were considered actionable. CIViC is also an expert-curated knowledgebase for interpretation of clinical relevance of both inherited and somatic variants in tumors [7]. To date, CIViC contains 3530 curated interpretations of clinical relevance for 3075 variants affecting 437 genes among which 2250 are treatment-related. The interpretations were curated from published literature, primarily over the last five years. Each interpretation was associated with one or two evidence records. While knowledgebases attempt to generate and evaluate evidence based on literature, it is hard to generalize individual findings from the literature. For example, even though CIViC curated 2250 are treatment-related evidence, only 16 assertions (knowledge generated from available evidence) regarding 9 genes and 13 mutations were confirmed and published.

Due to the heterogeneity of the Precision Oncology patient cohort, sample sizes for patients in the Precision Oncology literature are often small, and patient characteristics are unique. Therefore, it’s especially difficult to conduct large-scale clinical trial research or synthesize evidence into knowledge based on different Precision Oncology studies. In a real-world setting, not all targeted therapies are initiated despite the existence of actionable mutations. With the increasing accessibility of digital real-world data (RWD), using RWD to generate real-world evidence (RWE) can be an alternative, low-cost option to bridge the evidentiary gap between clinical research and practice. RWD is defined as data that is routinely generated or collected in the course of health care delivery [10]. Under the twenty-first century Cures Act, the FDA developed a program to evaluate the use of RWE to support approval of new indications for approved drugs or to satisfy long-term drug safety surveillance [11]. However, there are challenges to the effective utilization of RWD. One of the challenges includes a limited number of patients with a complete set of clinical characteristics within one institution. Therefore, it is desirable to increase the interoperability of RWD so that data can be integrated across multiple institutions. Large-scale consortiums such as The Cancer Genome Atlas (TCGA) [12] and Genomics Evidence Neoplasia Information Exchange (GENIE) [13] aim to create centralized databases to address this issue. Another approach to enhance interoperability is by using Wide Web Consortium (W3C) technologies, which provide a set of widely established standards [14]. The Resource Description Framework (RDF) is a recent W3C-recommended semantic web tool designed to standardize the definition and use of metadata [15]. It provides a data model that can be extended to address sophisticated ontology representation techniques [15]. In this paper, we describe our work that focused on increasing the interoperability of RWD by proposing a novel framework to capture RWD and then represent it using RDF. Based on RWD collected from an institutional oncology cohort, we generated a PO2RDF that can potentially be used for downstream analysis e.g., drug response monitoring, adverse event surveillance. We demonstrated two potential use cases of PO2RDF: (1) an examination of real-world associations between EGFR mutation and the prescription of targeted therapies. (2) An examination of differences in prognosis for lung cancer patients with/without TP53 mutations.

## Methods

**Fig. 1:**
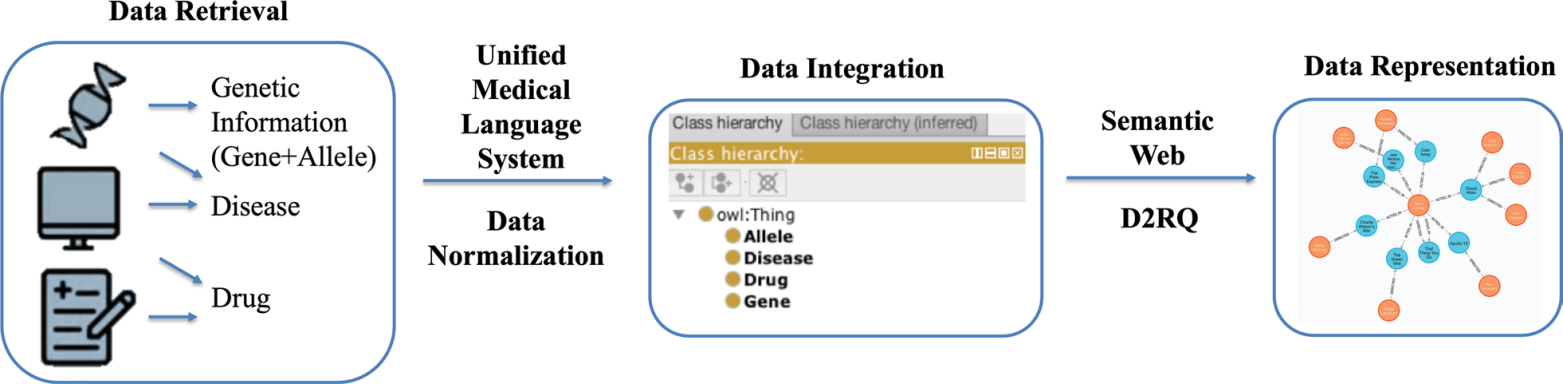
Workflow of RDF representation of real-world precision oncology data. (1) Data Retrieval: ‘patient’, ‘gene’, ‘variant’, ‘drug’, ‘disease’ information were retrieved from multiple data sources. (2) Data Normalization: raw data retrieved from multiple data sources were mapped to standardized terminologies including UMLS, etc. (3) data was integrated using a schema by Genetic Testing Ontology. 4) PO2RDF was generated using D2RQ

In this study, we generated an integrative and standardized data resource for RWD of Precision Oncology via multiple steps, (1) we semi-automatically collected RWD that belongs to key elements (e.g., gene, variant, disease, drug) in a previously proposed precision oncology knowledge model from EHRs; (2) we normalized the collected data using for further data integration; (3) we integrated collected data using a schema by Genetic Testing Ontology (GTO)[16], which captures the semantic meaning and semantic relations in the collected data; and (4) we generated PO2RDF using D2RQ[17]. The workflow performed in this study is shown in Fig. 1.

### Oncology cohort

Our cohort includes a total of 2,593 patients with Foundation Medicine tumor mutation tests (FoundationOne CDx and FoundationOne Heme). Foundation Medicine offers three different types of tumor panels and covers a range of 709 genes. All patients in the cohort have been granted research authorization and are aged above 18. This research project was approved by the Mayo Clinic Institutional Review Board (IRB# 13-009317) and was following the ethical standards of the responsible committee on human experimentation.

### Data retrieval

Based on the institutional oncology cohort, we semi-automatically collected RWD from genetic reports and electronic health records (EHRs). Patient IDs were linked to integrate data in genetic reports and EHRs by comparing (1) patient clinic number, (2) first and last name, and (3) date of birth. According to our previously proposed precision oncology knowledge model [18], three types of data elements were extracted: “genetic information” (“gene” + “variant”), “disease” and “drug”. Data sources that were used to retrieve three data elements are listed in Table 1. While “genetic information” was extracted from genetic reports only, “disease” and “drug” were retrieved from multiple sources, including genetic reports, a unified data platform (UDP), a structured clinical data warehouse of Mayo Clinic [19], and unstructured clinical notes. “Disease” was from both genetic reports and UDP. We extracted only cancer-related diagnosis information. When there is discordance between genetic report and UDP, we resort to genetic report as our gold standard. We combined “drug” information from UDP and an unstructured database. In this way, we assumed we had the most complete drug profile for each patient. For the extraction of “drug” concepts from unstructured clinical notes, we leveraged a dictionary from HemOnc.org [20] that curated comprehensive oncology medication knowledge. Sentences in patients’ clinical notes that mentioned drug concepts were extracted using a natural language processing (NLP) system called MedTagger [21]. MedTagger enables a series of NLP processes, including dictionary-based concept indexing, keyword mention lookup, and regular expression matching [22]. Both the drug brand name and chemical name were looked up and were normalized to chemical names.

**Table 1:** Data retrieval sources

	Gene	Variant	Disease	Drug
Genetic reports	Y	Y	Y	
UDP			Y	Y
Clinical notes				Y

### Data normalization

To facilitate data manipulation and integration, we performed data normalization on RWD extracted from multiple sources. In this study, we mapped “gene”, “variant”, “disease” and “drug” concepts to the Unified Medical Language System (UMLS) [23] via the batch process function offered by the MetaMap API[24]. The mapping results generated by the MetaMap include the UMLS preferred terms along with mapping scores. For variants that cannot be mapped to UMLS concepts, we manually normalized variant names to HGVS-nomenclature [25].

### Data integration

We leveraged schema from a previously developed ontology – GTO to integrated the collected RWD. GTO defined seven primary classes, namely ‘Diseases’, ‘Gene’, ‘Variant’, ‘Test’, ‘Phenotype’, ‘Risk’ and ‘Drug’ and the relationships among them [16]. We utilized four of GTO’s primary classes, namely ‘Diseases’, ‘Gene’, ‘Variant’ and ‘Drug’ and selected object properties include ‘AssociatedWithGene’ (Domain: ‘Disease’ and Range: ‘Gene’), ‘MayTreatedBy’ (Domain: ‘Disease’ and Range: ‘Drug’), ‘HasContraindicationWith’ (Domain: ‘Drug’ and Range: ‘Disease’), and ‘AssociatedWithVariant’ (Domain: ‘Gene’ and Range: ‘Variant’).

We inherited GTO’s data properties, especially identifiers that link to external knowledgebases such as Online Mendelian Inheritance in Man (OMIM) [26] and National Drug File Reference Terminology (NDF-RT) [27]. In addition, we added additional identifiers in the data property that link to other precision oncology knowledgebases, such as CIViC_Entrez_ID for identifying ‘Gene’ and CIViC_DOID for identifying ‘Disease’ in CIViC. We also incorporated drugs’ brand names (Brand_Name) and categories (Drug_Category) according to HemOnc as additional data properties. We also created a new data class ‘Patient’ to our data schema. The defined data properties for each class, along with some explanation are shown in Table 2.

**Table 2:** Description of data properties and related object properties

Class	Data property	Related object property
Patient	Patient_ID, Date_of_Birth, Race, Ethnicity, Sex, Death	HasMutGene, HasVariant, HasDisease, TreatedBy
Gene	Gene_Name, UMLS_CUI, OMIM_ID, CIViC_Gene_ID, OncoKB_Gene_ID, PharmGKB_Gene_ID	AssociatedWithGene, AssociatedWithVariant, MayTargetedBy
Variant	Var_Name, UMLS_CUI, ClinVar_ID, dbSNP_ID, CIViC_Var_ID, OncoKB_Var_ID,	AssociatedWithVariant
Disease	Disease_Name, UMLS_CUI, OMIM_ID, CIViC_DOID, OncoKB_Disease_ID, PharmGKB_Disease_ID, Stage_At_Diagnosis	AssociatedWithGene, MayTreatedBy, HasContraindicationWith
Drug	Drug_Name, Brand_Name, Drug_Category, UMLS_CUI, NUI (NDF-RT Unique Identifier), CIViC_Drug_ID, OncoKB_Drug_ID, PharmGKB_Drug_ID	MayTreatedBy, HasContraindicationWith, MayTargetedBy

‘Disease’ and ‘Gene’ relationships were considered valid for diagnosis up to one year before genetic tests. ‘Drug and ‘Gene’ associations (object properties) were considered valid for drug prescriptions up to one year after genetic tests and include targeted therapies only. ‘Disease’ and ‘Drug’ associations (object properties) were considered valid for drug prescriptions after disease diagnosis. For an individual patient, we only count each ‘Disease’ and ‘Drug’ associations once.

### PO2RDF generation

**Fig. 2:**
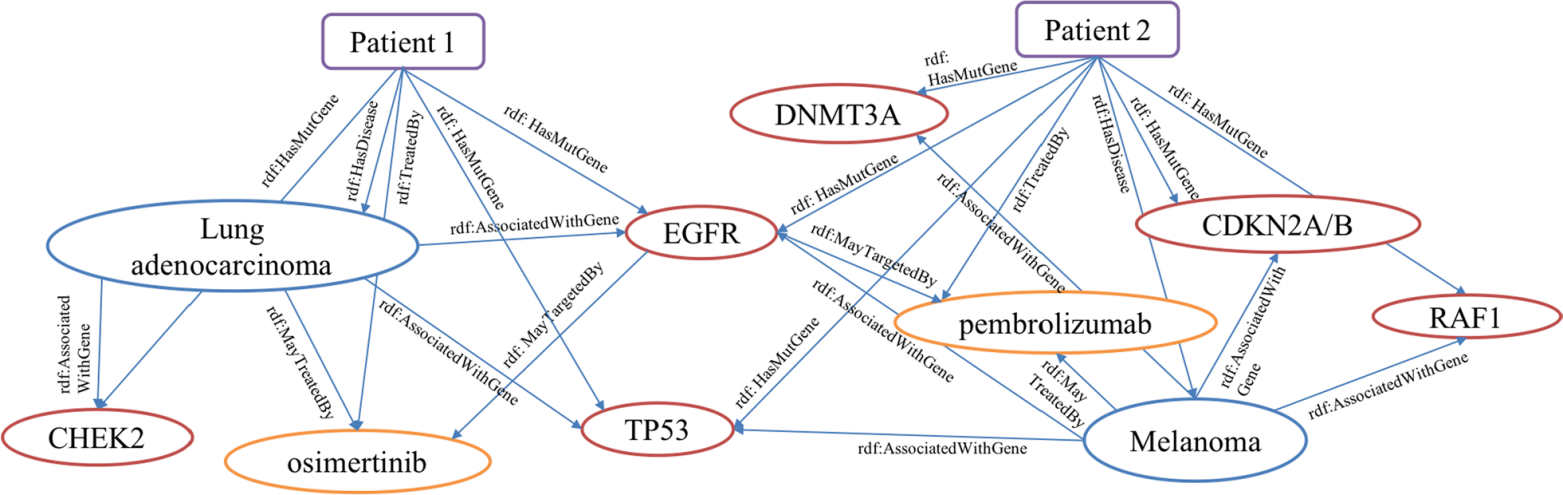
Example of RDF Representation of Two Patients’ Data (Purple square: ‘Patient’, Red circle: ‘Gene’, Orange circle: ‘Drug’, Blue circle: ‘Disease’). Patient 1 was diagnosed as lung adenocarcinoma, had variants in EGFR, TP53, CHEK2 gene and was prescribed Osimertinib after receiving the genetic report. Patient 2 was diagnosed as melanoma, had variants in EGFR, TP53, DNMT3A, CDKN2A/B, RAF1 gene and was prescribed Pembrolizumab after receiving the genetic report

For the PO2RDF generation, we applied D2RQ, which transforms data in the relational database to RDF. The mapping tool of D2RQ creates a default mapping file by analyzing the schema of an existing database. To map our data to the GTO schema, we manually customized the mapping file accordingly. The data is then published in RDF through the D2RQ server and can be queried via a D2RQ SPARQL endpoint. We also took an RDF dump from D2RQ into Virtuoso [28] to run federated queries. Figure 2 shows detailed RDF representation of two patients. “Variant” elements were not represented due to space limit.

### Use cases

To demonstrate the usability of PO2RDF, we retrieved triples involving ‘Gene’ and ‘Drug’ from PO2RDF. We then performed association rule analysis [29] to evaluate the significance of real-world associations between mutated genes and selected oncology drugs. First, we examined drugs associated with the gene “EGFR”, which is most commonly identified and targeted in lung cancer [30], colorectal cancer [31, 32] and melanoma [33] patients. EGFR inhibitors were initially approved to treat non-small cell lung cancer (NSCLC) and appear to be most effective in patients with adenocarcinoma histology [30]. Even though current FDA drug approved indications for EGRF inhibitors are mostly for NSCLC, they are also used off-label [31–33] for other cancers in real-world settings. Therefore, the results from our association analysis could potentially provide RWE to clinicians and the FDA regarding the real-world utility of targeted therapies—especially any deviations from guidelines or drug labels. Second, we examined differences in prognosis for lung cancer patients with/without TP53 mutations at different stages using survival analysis. The index date was the disease diagnosis date retrieved from UDP. Most mutations in TP53 lead to the uncontrolled cell proliferation and inability to trigger apoptosis in cells [34]. Across multiple cancer types, individuals with TP53-mutated cancers have consistently been shown to have a lower response rate to conventional chemotherapy and shorter survival [35]. Therefore, the results from our survival analysis should align with currently agreed knowledge to demonstrate the utility of PO2RDF for future survival analysis.

We calculated the confidence of each {“Drug”, “EGFR”} transaction (Eq. 1). The support of X with respect to a group of transactions T is defined as the proportion of transactions t in the dataset which contains the item X (Eq. 2). Each individual patient was considered as one transaction (t). Our cohort of 2593 patients were considered as the total transaction set T.

1 $$\mathrm{confidence }\,\left(\mathrm{X},\mathrm{ Y}\right)= \frac{\mathrm{support }\,(\mathrm{X }\cup \mathrm{Y})}{\mathrm{support }\,(\mathrm{X})}$$

2 $$\mathrm{support }\,\left(\mathrm{X}\right)= \frac{|\{\mathrm{t}\in \mathrm{T};\mathrm{X}\in \mathrm{t}\}|}{|\mathrm{T}|}$$

## Result

### Oncology cohort

**Fig. 3:**
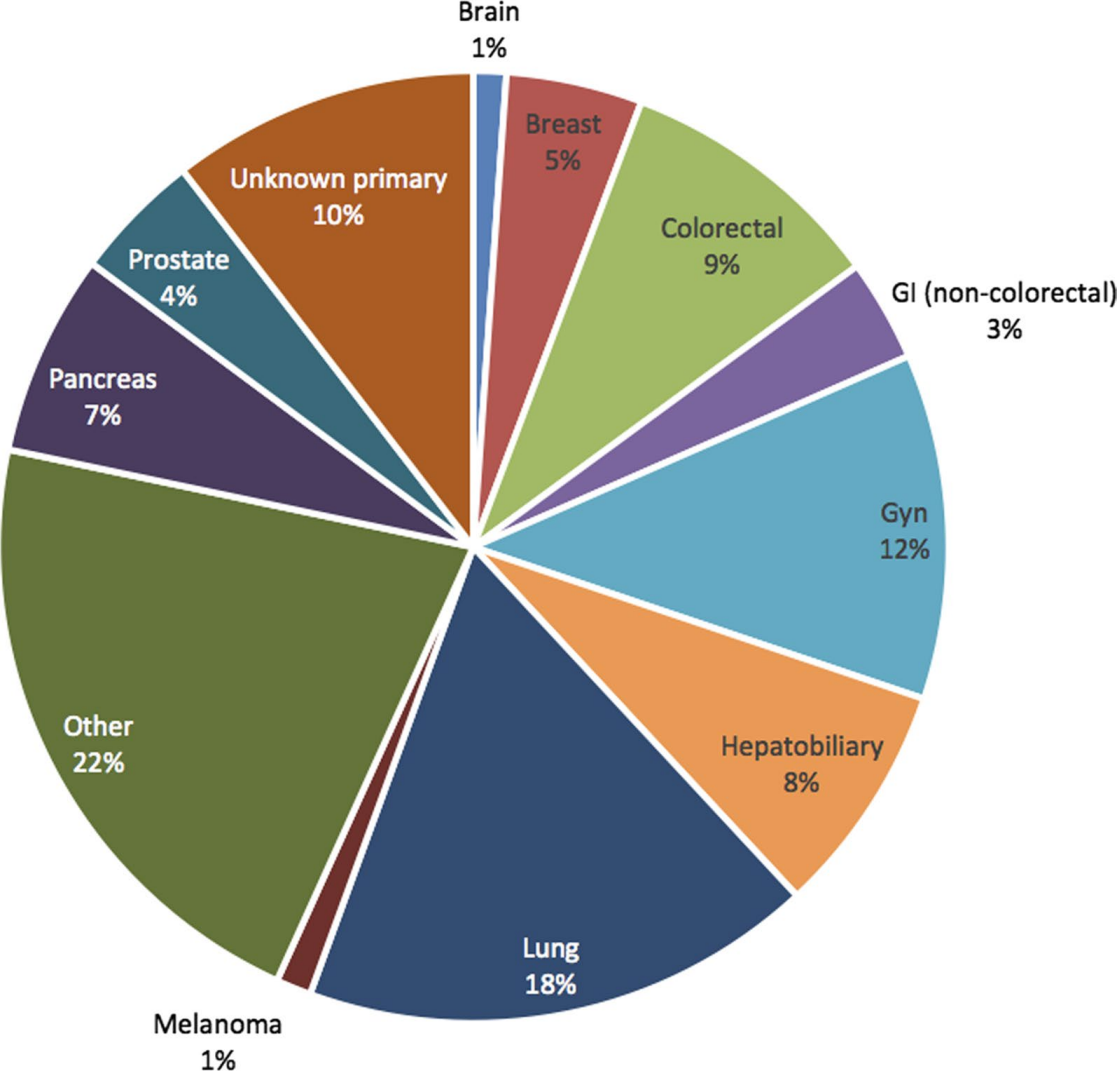
Distribution of Major Cancer Type in the Institutional Oncology Cohort (N = 2593)

We have constructed an oncology cohort of 2593 (authorized, age ≥ 18) oncology patients with clinically provided genetic reports. Date of report receipt range from January 2016 to June 2020. Only treatment initiated after report receipt date was included in our analysis. Shown as Fig. 3, this cohort consists of 10 primary types of tumors and is representative of the diversity of patients seen at a dedicated cancer center. As a note, unknown primary cancer cases encompass 10% of the cohort, which indicates the complexity of cases received at Mayo Clinic. In UDP, we were able to retrieve diagnosis codes of 1193 (46%) patients, among which we were able to identify cancer related diagnosis for 658 patients and 176 received their primary cancer diagnosis at Mayo. This again indicate that heterogeneity of patient population treated at Mayo Clinic—a significant proportion of patients might be referral patients. Thus, combining multiple clinical data sources, especially unstructured clinical notes is crucial to comprehensive RWD capturing. Patient demographic distribution is shown Table 3.

**Table 3:** Cohort demographic distribution

Characteristic	Cohort (n = 2593)
Average age at initial diagnosis at Mayo Clinic	58
Average age at first test	62
Sex (% female)	51.4%
Race (% white)	88.7%
Ethnicity (% hispanic)	3.5%

### Data normalization and integration

To represent PO2RDF in a normal form for further data integration, we mapped individual terms in four classes to UMLS. Table 3 lists the summary of concepts in all four classes. We randomly selected one hundred mapping results for each type of term and manually reviewed the mapping results. According to our evaluations, there are no incorrect mappings for one hundred ‘Drug’ and ‘Variant’ terms, but there is one incorrect mapping among one hundred ‘Gene’ terms caused by ambiguity with another disease abbreviation term and two incorrect mappings among one hundred ‘Disease’ terms caused by substring matching. Despite that ‘Variant’ mappings have been largely accurate, it suffers from huge missingness mainly due to variations in nomenclature between genetic report and UMLS terminology sources. Table 4 lists statistical overview for the final data.

**Table 4:** Statistical results for data collection

	Total number of occurrences	Total number of UMLS-identifiable occurrences	Unique concepts	Unique UMLS-identifiable concepts
Gene	17,100	17,018 (99.5%)	417	415
Variant	16,196	3,158 (19.5%)	5497	285
Disease	109,030	107,106 (98.2%)	8449	8102
Drug	249,995	249,853 (99.9%)	389	368

### PO2RDF generation

**Fig. 4:**
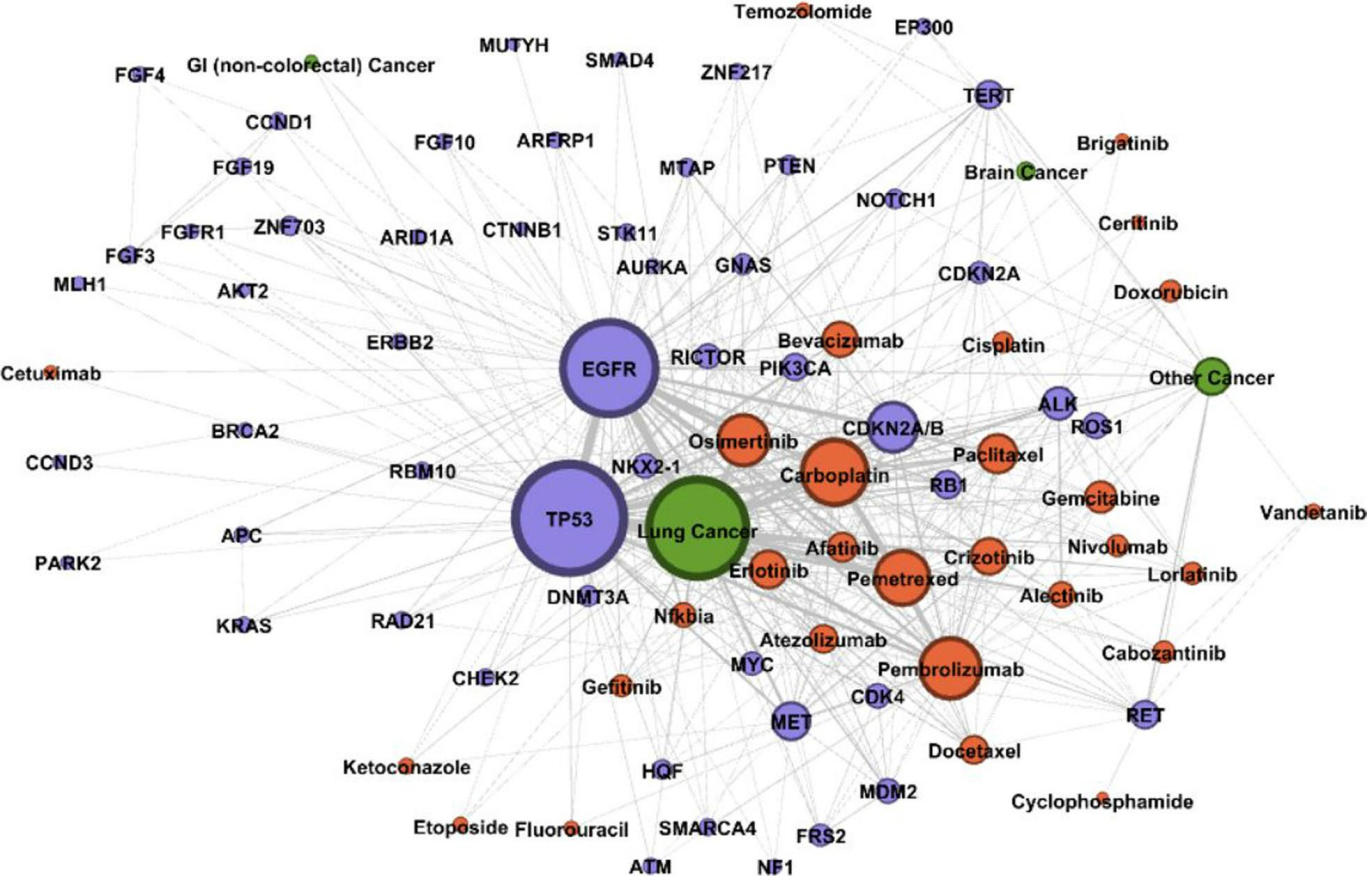
Visualization of RDF Representation of Real-world Associations Among “Gene” (Purple), “Disease” (Green), and “Drug” (Orange) in Precision Oncology. Node size represents the degree of each node (unique number of co-occurrence). Edge thickness represents the weight of each edge (total counts of co-occurrences)

There are total 2,309,014 triples contained in the PO2RDF. Among them 32,815 triples are related to Gene, 34,695 triples are related to Variant, 8787 triples are related to Disease, 26,154 triples are related to Drug. Table 5 include an example SPARQL query and retrieved pertinent information centered on “EGFR”, shown in the “SPARQL Query” column. Specifically, we are searching for related diseases and available targeted drugs, shown in the “Results” column in the Table 5 (for ‘Disease’ and ‘Drug’, only listed top five returned values). An example of data representation of precision oncology evidence from real-world data can be found in Fig. 4. “Variant” elements were not represented due to space limit. We can see from Fig. 4 that drugs most associated with “lung cancer” are “carboplatin”, “osimertinib”, “pemetrexed”, “gefitinib”, “afatinib”, “erlotinib” and “crizotinib. Genes most associated with “lung cancer” include “TP53”, “EGFR”, “CDKN2A/B” and “MET”. However, a graph visualization mask it hard to see a tertiary association e.g., drugs association with lung cancer with EGFR mutations. Thus, an RDF structure enables more efficient query and visualization of complicated graph database.

**Table 5:** SPARQL query to extract EGFR related information

SPARQL query	Results
SELECT distinct ?Gene ?property ?hasValue WHERE { ?Gene a po2rdf:Gene. FILTER regex(str(?Gene), "EGFR") ?Gene ?property ?hasValue }	Gene_Name: EGFR. UMLS_CUI: C1414313. OMIM_ID: 131550. CIViC_Gene_ID: 1956. OncoKB_Gene_ID: 2. PharmGKB_Gene_ID: PA7360 Disease_Name: 1. Lung cancer, 2. Colorectal cancer, 3. Melanoma, 4. Esophagus adenocarcinoma, 5. Glioma Drugs_Name: 1. Gefitinib, 2. Osimertinib, 3. Afatinib, 4. Erlotinib, 5. Dacomitinib Patient_ID: 3, 15, 21, 44, 65, 73…

### Use case

**Fig. 5:**
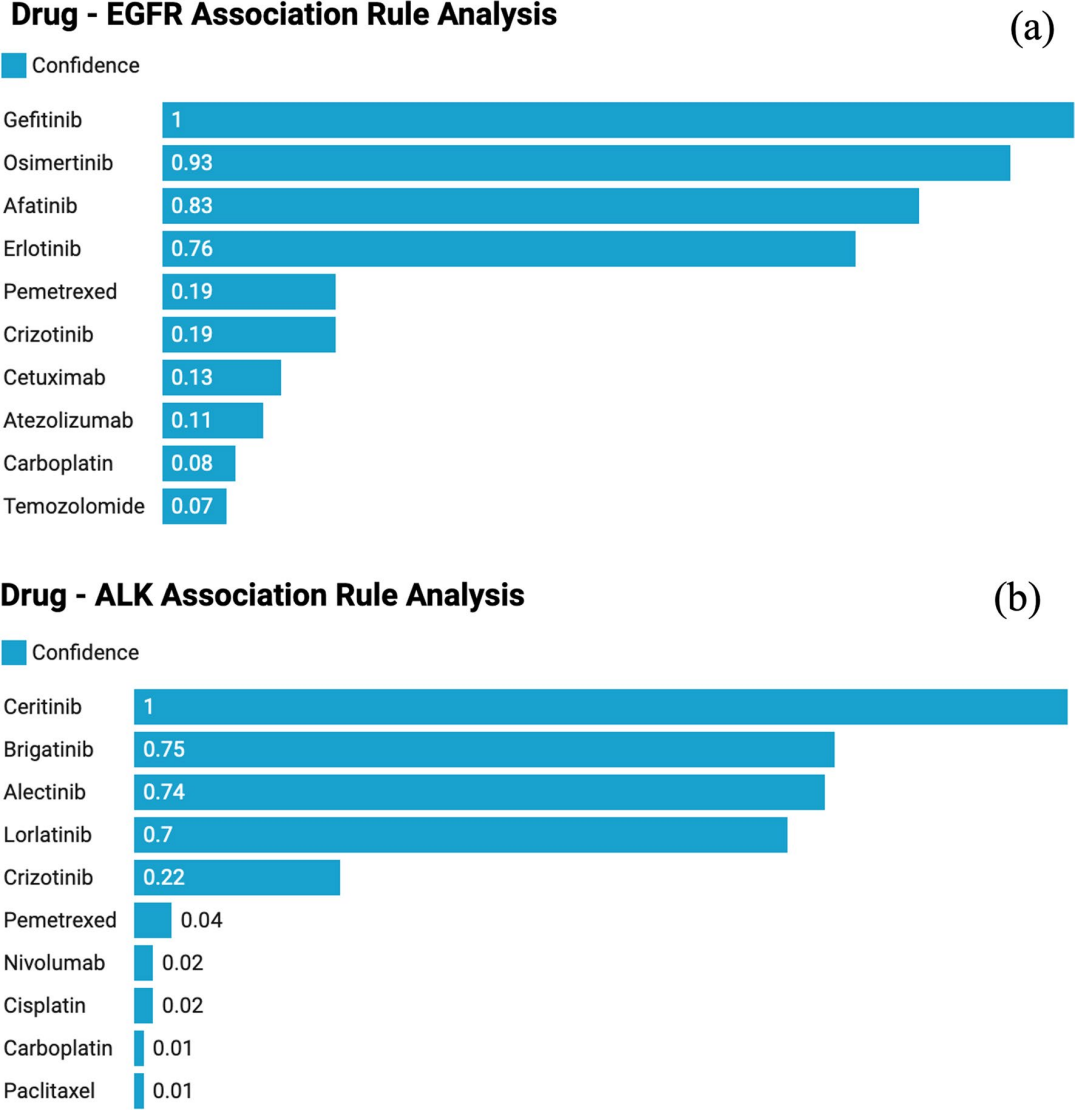
Association rule analysis results (confidence) regarding drug—**a** EGFR and **b** ALK mutation Associations

The result from association analysis is shown in Fig. 5. The top ten EGFR-associated (measured by “confidence”) drugs were listed and they are “gefitinib”, “osimertinib”, “afatinib”, “erlotinib”, “pemetrexed”, “crizotinib”, “cetuximab”, “atezolizumab”, “carboplatin”, and “temozolomide” [36–43]. The top four drugs are all specific EGFR tyrosine kinase inhibitors (TKIs) and they all have a high “confidence” value of association. Importantly, association rule analysis identified all the EGFR TKIs that are in clinical use in the US. “Confidence” value for “pemetrexed” is significantly lower than the top four, reflecting that “pemetrexed” is not a targeted therapy for EGFR mutated cancers. “Pemetrexed” is a cytotoxic chemotherapy drug that can be used to treat mesothelioma and non-small cell lung cancer. “Crizotinib” is also not an EGFR-targeted therapy. Rather, it is effective in NSCLC driven by activating genomic alterations in “MET”, “ALK” and “ROS1”. Interestingly, although the confidence value for crizotinib and pemetrexed is lower than for specific EGFR TKIs, it is still higher than for carboplatin. This observation reflects the use of crizotinib in combination with EGFR TKIs to treat patients with mutant EGFR positive lung cancer that have developed resistance to EGFR inhibition by acquiring a high MET gene copy number. Additionally, pemetrexed is approved for patients with non-squamous but not for squamous NSCLC, a population enriched in EGFR mutations compared to the population of cancer patients who qualify for treatment with carboplatin. “Cetuximab” is an EGFR inhibitory antibody but it does not show high specificity to EGFR mutations [44]. Overall, the order of confidence values mirrors the prevalence of EGFR mutations in the groups of patients with NSCLC who receive the corresponding drugs. Similarly, association analysis for ALK shown in Fig. 5b, correctly assigned much higher confidence values for all TKIs with ALK specificity, namely crizotinib, lorlatinib, alectinib, brigatinib and ceritinib compared to chemotherapy drugs and immune check point inhibitors that are prescribed in an ALK agnostic manner. The confidence value for crizotinib is lower than for the other ALK TKIs, as crizotinib can also be prescribed to patients with NSCLC and activating genomic alterations in MET or ROS1.

**Fig. 6:**
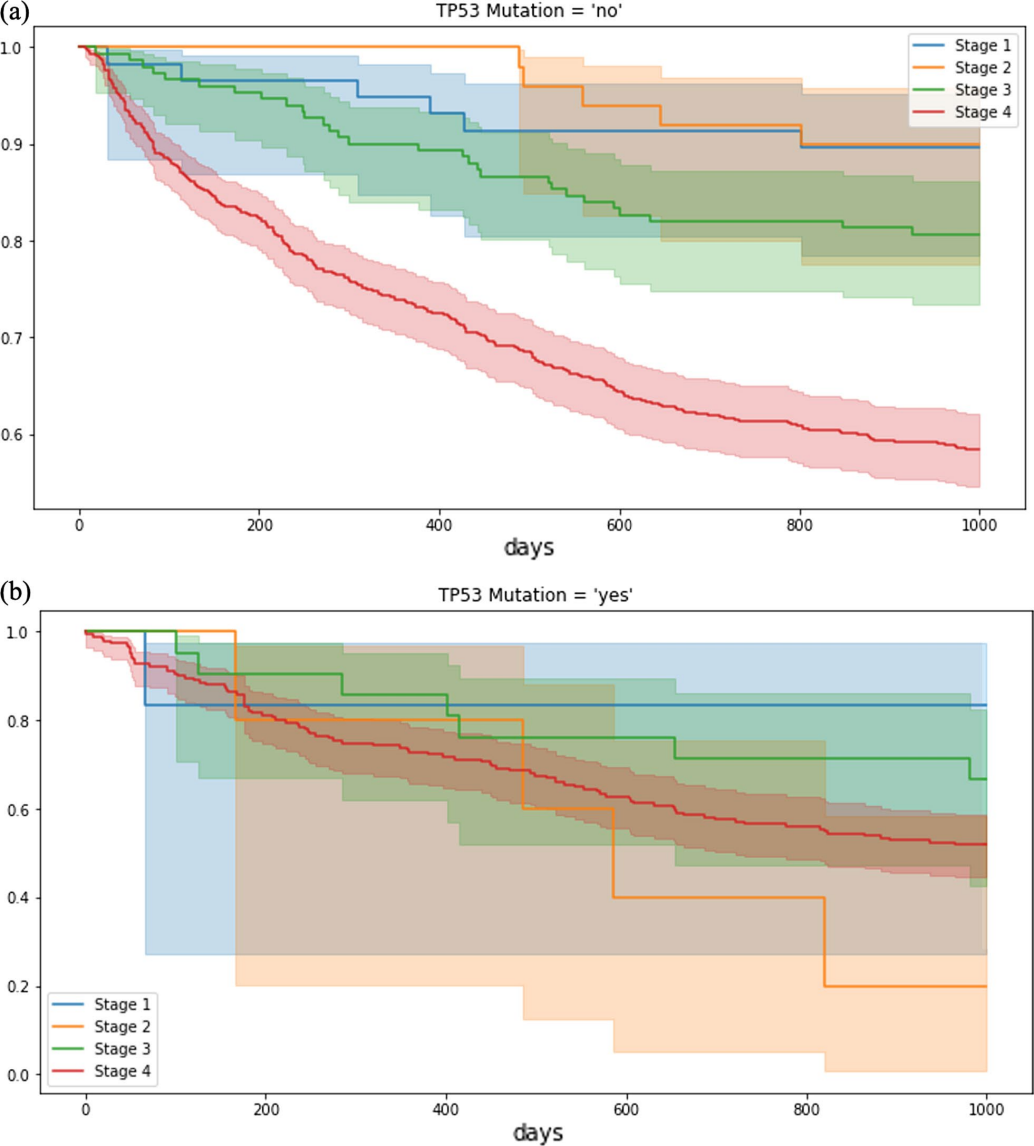
Survival analysis for lung cancer patients **a** with or **b** without TP53 mutation

Results from survival analysis are shown in Fig. 6. It is clearly shown in the figure that patients with TP53 mutations have shorter durations of survivals especially for patients at advanced stages (stage III and IV). Therefore, the results demonstrate a potential use of PO2RDF to answer more clinically relevant questions regarding drug effectiveness with the existence of certain variants.

## Discussion and future work

In this study, we introduced a novel precision oncology RDF data resource by integrating heterogeneous information about patients from multiple data sources. Potential use of PO2RDF has been demonstrated in the use case. For example, SPARQL queries could facilitate retrieval of comprehensive information regarding genetic mutations and treatment choices by searching the PO2RDF and other relevant and linked knowledgebases. Moreover, with survival available, we could utilize PO2RDF to answer more clinically relevant questions regarding drug effectiveness with the existence of certain variants. Additional data analytics also demonstrated the potential to use information in PO2RDF for treatment recommendation given a mutated gene. In addition to our demonstrated use case, RDF provides a powerful framework for integrating external data sources e.g., knowledgebases, data from other institutions. Through actively feeding new RWD into PO2RDF, PO2RDF can also serve as a data foundation for a learning health system [45, 46] and can ultimately support the development of clinical decision support systems (CDSS) in Precision Oncology practices. If adopted by several institutions, PO2RDF could serve as a tool to enhance interoperability and promote data sharing among participating institutions.

However, there are still challenges in the data normalization phase—even though mapping data in classes ‘Gene’, ‘Disease’ and ‘Drug’ to UMLS achieved a high performance, mapping data in ‘Variant’ suffered from low coverage (19.5%). There are two reasons that potentially contribute to the low coverage. (1) In UMLS, variant terms mainly come from two sources: OMIM and the National Cancer Institute (NCI). While SNVs have a relatively standardized nomenclature, deletion, insertion, loss, duplication and rearrangement are recorded variably in OMIM, NCI and genetic reports. For example, the genetic report variant “CDKN2A deletion exon 1” will be recorded as “CDKN2A, EXON 1-BETA DEL” in OMIM or simply “CDKN2A Gene Deletion” in NCI. Therefore, it is difficult to extract through regular expression without further normalization. In future work, tools that normalize variant nomenclature to UMLS can be developed to address this unmet need. (2) Both OMIM and NCI have limited records of variants. For example, most frameshift and splice site mutations are not documented in them. A great percentage of fusions cannot be found or can only be mapped partially: “CD74-ROS1 fusion” in genetic reports can only be mapped to “ROS1 Fusion Positive”. Therefore, incorporating more comprehensive variant knowledgebases such as ClinVar [9] and COSMIC [47] into UMLS is desirable. We also propose to use a structured data entry system supported by clinical terminology in a clinical setting for genetic information documentation. This could save time for data input, encourage documentation of genetic information and ensure high quality data capture.

One of the limitations of our PO2RDF network is that relationships between ‘drug’, ‘disease’, and ‘gene’/‘variant’ are only associative. To confirm a causal relationship will require additional information to be collected from EHRs or other knowledgebases. In the future, we plan to incorporate knowledgebase relationships into the RDF so that associative relationships mined from EHRs can be further validated. We also plan to expand data properties by adding temporal information to each data element. With temporal information, we will be able to make less biased associations between data elements and discover any dynamic pattern changes in the network that may be reflective of disease progression or practice change due to regulatory changes. RDF enables a mathematical and computable representation of relationships between data elements. Therefore, more downstream analysis can be achieved by formatting the database into an RDF structure. With a more complete RDF graph, we can apply advanced graph mining [48] technologies such as node2vec [49] to discover hidden patterns within the PO2RDF network, which could potentially provide insights to drug repurposing.

## Conclusion

In conclusion, our work proposed to use RDF to organize and distribute clinical RWD that is otherwise inaccessible externally. Our work serves as a pilot study that will lead to new clinical applications and could ultimately stimulate progress in the field of precision oncology.

## Data Availability

The data used in this study cannot be shared publicly because of the patient health information included in the texts. Please contact the corresponding author for future data access.

## Abbreviations

RDF: Resource description framework
PO2RDF: Precision oncology to RDF
FDA: Food and Drug Administration
RWD: Real-world data
RWE: Real-world evidence
TCGA: The cancer genome atlas
GENIE: Genomics Evidence Neoplasia Information Exchange
W3C: Wide web consortium
GTO: Genetic testing ontology
EHRs: Electronic health records
UDP: Unified data platform
NLP: Natural language processing
UMLS: Unified medical language system
OMIM: Online Mendelian inheritance in man
NDF-RT: National drug file reference terminology
NSCLC: Non-small cell lung cancer
TKIs: Tyrosine kinase inhibitors
CDSS: Clinical decision support system
NCI: National Cancer Institute
